# Optimizing the anesthetic care of patients with aromatic l‐amino acid decarboxylase deficiency

**DOI:** 10.1111/pan.15025

**Published:** 2024-10-22

**Authors:** Megha K. Kanjia, Edmund H. Jooste, Melissa Illig, Jennifer Neifeld Capps, Christoph Eisner, Shou Zen Fan, Jerzy Lenarczyk, Rafał Wojdacz

**Affiliations:** ^1^ Texas Children's Hospital Houston Texas USA; ^2^ Baylor College of Medicine Houston Texas USA; ^3^ Duke University School of Medicine Durham North Carolina USA; ^4^ Boston Children's Hospital Boston Massachusetts USA; ^5^ Heidelberg University Hospital Heidelberg Germany; ^6^ National Taiwan University Hospital Taipei Taiwan; ^7^ University Center for Women's and Newborn Health Warsaw Poland

**Keywords:** AADC deficiency, anesthesia, gene therapy, perioperative management

## Abstract

Aromatic l‐amino acid decarboxylase (AADC) deficiency is a rare autosomal recessive disorder that results in a lack of the monoamine neurotransmitters dopamine, serotonin, norepinephrine, and epinephrine. Patients present with a wide spectrum of symptoms, including motor and autonomic dysfunction, hypotonia, and developmental delay, often before the age of one. Until recently, treatment options were limited to symptom control, but the recent approval of the first gene therapy for AADC deficiency in Europe and the UK has provided an alternative to treating symptoms for this disease. Eladocagene exuparvovec is a one‐time gene therapy, administered bilaterally to the putamen by magnetic resonance imaging‐guided stereotactic neurosurgery. While administration of the gene therapy itself is minimally invasive, the anesthetic management of patients with AADC deficiency is challenging due to the absence of sympathetic regulation secondary to the lack of adrenergic neurotransmitters. Optimal anesthetic management requires an understanding of the complex and heterogeneous nature of the disease. Hemodynamic instability, temperature dysregulation, and hypoglycemia are of primary concern, but there are also challenges regarding intravenous access and airway management. A thorough preoperative assessment is essential and should be guided by the patient's history. Advanced planning is necessary regarding the timing of the procedure schedule and operative plan; meticulous preparation, simulation for the operating room, as well as communication with all perioperative staff members, are crucial. Intraoperatively, utmost care must be taken to protect the skin, maintain body temperature, and to prepare for inotropic and/or glycemic support as needed. Postoperative intensive care management is necessary for consideration of postoperative extubation and provision of supportive care. With careful planning, preparation, and vigilance, patients with AADC deficiency can safely undergo anesthesia.

## 1 INTRODUCTION TO AADC DEFICIENCY

Aromatic l‐amino acid decarboxylase (AADC) deficiency is a rare autosomal recessive disorder of neurotransmitter biosynthesis.^1^, ^2^, ^3^ Although reports indicate that 43% of cases are found in the Asian population due to a founder variant, AADC deficiency has been diagnosed in patients of different ethnicities worldwide.^1^, ^2^, ^4^, ^5^ Estimating its prevalence remains challenging, and while globally less than 350 patients were previously reported in the literature, the true incidence is likely to be greater than generally recognized.^1^, ^6^, ^7^

**FIGURE 1:**
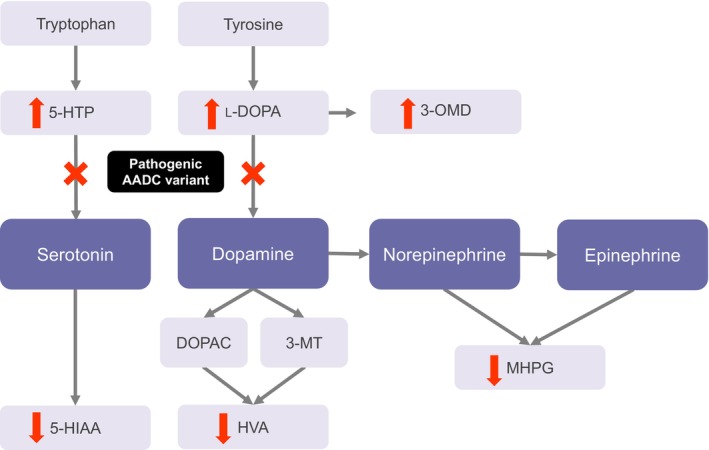
Loss of AADC enzyme activity leads to altered catecholamine and serotonin synthesis, with concomitant changes in the levels of neurotransmitter precursors and metabolites. 3‐MT, 3‐methoxytyramine; 3‐OMD, 3‐O‐methyldopa; 5‐HIAA, 5‐hydroxyindoleacetic acid; 5‐HTP, 5‐hydroxytryptophan; AADC, aromatic l‐amino acid decarboxylase; DOPAC, 3,4‐dihydroxyphenylacetic acid; HVA, homovanillic acid; l‐DOPA, l‐3,4‐dihydroxyphenylalanine; MHPG, 3‐methoxy‐4‐hydroxyphenylglycol.

AADC deficiency is caused by pathogenic variants in the dopa decarboxylase (*DDC*) gene, which encodes the enzyme required for the final step of the monoamine neurotransmitter synthesis pathway.^8^ Loss of AADC activity leads to a deficiency of serotonin and dopamine, and consequently of epinephrine and norepinephrine^4^, ^9^ (Figure 1). As a result, patients with AADC deficiency have complex motor, behavioral, cognitive, and autonomic impairment, with symptom onset usually occurring within the first year of life.^1^, ^5^, ^10^ Initial symptoms may be nonspecific and include feeding difficulties, hypotonia, and developmental delay. However, these can vary greatly and may overlap with other common neurological symptoms, often resulting in misdiagnosis.^1^, ^2^, ^11^ More specific symptoms include oculogyric crises, dystonia, and autonomic dysfunction, the latter manifesting as nasal congestion, abnormal sweating/temperature homeostasis, excessive drooling, stridor secondary to laryngomalacia or tracheomalacia, hypotension, and bradycardia. Other characteristic symptoms include a failure to thrive, irritability, excessive crying, apnea, gastrointestinal disturbances, and hypoglycemia.^1^, ^2^, ^5^, ^12^, ^13^, ^14^ The phenotypic spectrum can range from a relatively mild to a very severe phenotype. However, most patients (80%) are classified with a severe phenotype and are fully dependent with no or very limited attainment of developmental milestones.^1^, ^5^

## 2 TREATMENT AND MANAGEMENT OF AADC DEFICIENCY

### 2.1 Pharmacological treatment

Until very recently, treatment options for AADC deficiency were pharmacologically based, aimed at: enhancing residual AADC enzyme activity (pyridoxal phosphate), potentiating dopamine receptor action (dopamine agonists), or slowing down the catabolism of serotonin and dopamine (monoamine oxidase inhibitors).^1^, ^2^, ^14^, ^15^ Other agents have also been used for autonomic symptoms (anticholinergics), sleep disorders (melatonin), and dystonic or ocular crises (benzodiazepines).^1^ Most patients have variable responses to these treatments, but while they may help alleviate selected symptoms, they do not address the underlying cause of the disease nor fundamentally restore neurotransmitter levels to adequately improve function.^1^, ^2^ Moreover, ergot‐based dopamine agonists such as bromocriptine are associated with cardiac fibrosis, placing patients with AADC deficiency at greater risk of cardiac instability.^1^

### 2.2 Gene augmentation therapy

Gene augmentation therapy has the potential to treat the underlying defect in AADC deficiency by delivering a functional gene to target cells without removing or replacing the mutant gene.^16^ Eladocagene exuparvovec is a novel therapy of this type for patients with severe AADC deficiency with a genetically confirmed diagnosis and has been available in the European Union and the United Kingdom since 2022.^17^ The product consists of a recombinant adeno‐associated virus serotype 2 vector containing the complementary human *DDC* gene and is administered as a one‐time therapy into the putamen via bilateral infusion.^18^

The efficacy and safety of eladocagene exuparvovec have been demonstrated in three clinical studies involving 30 patients, aged 19 months to 8.5 years, mostly from Taiwan. At baseline, all patients had no head control and were unable to sit or stand. Treatment with eladocagene exuparvovec increased putaminal dopamine production and provided rapid and clinically meaningful improvements in motor and cognitive function compared with baseline, which were sustained for many years after therapy.^18^ These improvements included achievement of motor milestones such as full head control, the ability to sit unassisted or stand, and in some patients the ability to walk freely without assistance by the end of their follow‐up period.^18^ Significant improvements in language and cognitive scores, symptoms (mood, sweating, temperature homeostasis, and oculogyric crises), body weight/growth, and caregiver quality of life were also observed during follow‐up for more than 5 years.^18^ Eladocagene exuparvovec was generally well tolerated and demonstrated a favorable safety profile. The most frequently reported adverse events were pyrexia during the immediate postoperative period and transient dyskinesia occurring around 1 month after surgery.^18^, ^19^ The safety of eladocagene exuparvovec was also evidenced by long‐term follow‐up (up to 9 years) of patients following surgery; no serious side effects related to the gene therapy were reported.^18^

Apart from eladocagene exuparvovec, other gene augmentation therapies targeting the putamen or midbrain are being investigated in AADC deficiency and show similar clinically meaningful responses.^6^, ^20^ Thus, gene therapy as a causal treatment is likely to change the future therapeutic landscape of AADC deficiency, meaning that healthcare professionals must be prepared for its implementation in clinical practice. Effective treatment relies on the delivery of the gene therapy into the brain parenchyma by stereotactic neurosurgery.^6^, ^18^, ^20^ Anesthesiologists play a critical role throughout the perioperative period to ensure successful delivery of the gene therapy treatment to eligible patients.

## 3 ANESTHETIC CHALLENGES IN AADC DEFICIENCY

Anesthetic management of patients with AADC deficiency can be challenging given the dependence of the physiological responses to dopamine. Hemodynamic instability, temperature variability, and hypoglycemia due to sympathetic dysfunction but intact parasympathetic activity are common issues. Additionally, there may be challenges regarding intravenous (IV) access, apnea, and airway support.^1^, ^21^, ^22^ Since anti‐dopaminergic, anti‐adrenergic, and anti‐serotonergic agents can worsen the symptoms of monoamine neurotransmitter deficiency,^21^ care must be taken with medications that specifically interact with these pharmacologic profiles. Moreover, in this patient population, phenylephrine can cause profound bradycardia, while ephedrine has diminished activity due to its indirect mechanism of action.^21^ Vigilance, along with an understanding of the common symptoms and issues, is therefore necessary to ensure safe delivery of anesthesia in patients with AADC deficiency.

Information on the optimal perioperative care of pediatric patients with AADC deficiency is currently very limited. Together with a case series, this paper aims to summarize the best practice recommendations and pragmatic advice in the context of intracerebral gene therapy treatment. The recommendations outlined may be adapted for other rare inherited neurotransmitter disorders and can also be applied to other procedures involving anesthesia.

### 3.1 Case series

Twenty patients with AADC deficiency, ten of whom were aged between 5 and 15 years, six between 15 and 18 years, and four over 18 years of age, underwent intracerebral gene therapy treatment at the Mazowiecki Hospital Bródnowski, Warsaw, Poland. In preparation, patients had IV induction anesthesia with propofol and remifentanil prior to tracheal intubation. Non‐depolarizing agents were used for muscle relaxation to facilitate intubation and optimal surgical conditions, but reversal of the effects of non‐depolarizing drugs were not required for this group of patients. Immediately after anesthetic induction, central venous access was established as gaining peripheral access was often difficult in these patients. Arterial catheterization enabled assessment of blood gases and direct measurement of blood pressure, while a urine catheter allowed close monitoring of urine output during the procedure. An inherent element of the surgical procedure was the qualitative assessment of cerebrospinal fluid (CSF). Therefore, immediately before the start of surgery, CSF was collected for monoamine neurotransmitter analysis. However, deformation of the chest and spine (due to the inability to move independently and reduced muscle tone), particularly in older patients, often made CSF collection via lumbar puncture difficult. During the surgery, cardiac activity, blood pressure, oxygen saturation, glycemia, body temperature, and diuresis were routinely monitored. There were no reports of hypothermia; however, hypoglycemia occurred in two patients (~50 mg/dL), and an increase in body temperature above 38°C, and/or hemodynamic instability unresponsive to dopamine, were frequently recorded. In such cases, hypoglycemia was compensated for with 40% glucose solution, hyperthermia corrected by lowering the ambient temperature, and hypotension mitigated by epinephrine or norepinephrine infusions. After surgery, local anesthesia (0.5% bupivacaine solution) was applied to the surgical field; paracetamol, metamizole, and, if necessary, buprenorphine were administered for pain relief. Patients were allowed to emerge from anesthesia with tracheal extubation taking place in the operating room prior to transfer to the post‐anesthesia care unit. Patients underwent follow‐up magnetic resonance imaging (MRI) of the brain 24–48 h post‐surgery under general IV anesthesia with fentanyl and propofol; no anesthetic complications were reported during this time.

Typically, the neurosurgical procedure lasted between 6 and 10 h. The main challenge encountered was overheating during surgery leading to vasodilation, decrease in systemic vascular resistance, and hypotension. Despite existing recommendations in the literature, administration of dopamine up to 10 mcg/kg/min was ineffective in the first patient. Thereafter, epinephrine and/or norepinephrine (0.01–0.03 mcg/kg/min IV) was used in subsequent patients with hemodynamic instability with greater success. Another challenge was the lack of MRI‐compatible equipment to measure body temperature accurately. However, this was overcome by using a liquid crystal strip thermometer fixed to the upper abdomen, which turned out to be a simple and effective method.

## 4 ANESTHETIC MANAGEMENT IN AADC DEFICIENCY

The anesthetic management of patients with AADC deficiency should be individualized given the rarity, complexity, and heterogeneity of the disease. A comprehensive plan covering the entire perioperative period, which takes into consideration the patient's profile, any technical limitations, and the proper handling of potential issues, is recommended.

### 4.1 Preoperative assessment

Emphasis should be placed on preoperative assessment and consultation to preempt any potential issues that may arise during the intraoperative or postoperative periods.

#### 4.1.1 Good communication

The patient's history should be obtained from the caregivers to help better understand the baseline characteristics, such as the general level of agitation, extent of apnea, and degree and normal management of temperature fluctuation. This detailed history will help determine what intervention, if any, is required and when it should be applied. For example, many patients display what appears to be agitation or anxiety at baseline. This perceived agitation is part of the disease process due to the lack of serotonin. Since there are no medications to effectively treat such symptoms in patients with AADC, anxiolysis may not necessarily be required prior to procedures even though the patient may appear to be anxious in the preoperative holding area. Frequent and significant fluctuations in temperature may be common in these patients necessitating the use of blankets (placement or removal) or other methods to moderate temperature, according to the history given by caregivers.

Procedural, anesthesia, and critical care teams should be in communication regarding the perioperative plan. It is important that all staff involved in the perioperative care of the patient are made aware of the patient's clinical characteristics and specific personal care requirements. This may include what medications to avoid, how best to position the patient, the risk of reflux/aspiration, and the need for ultrasound‐guided line placement.

#### 4.1.2 Airway assessment

Of critical importance is an assessment of the patient's airways to avoid unanticipated difficulties with airway management. Patients with AADC deficiency may have structural abnormalities or other respiratory features that make airway securement challenging. Reports in the literature indicate that some patients in this population have tracheomalacia or laryngomalacia, which could affect endotracheal tube size selection or airway securement.^23^ Excessive salivation may be problematic but can be controlled with glycopyrrolate and should be monitored during longer procedures given the risk for unplanned extubation. Other opportunities to secure an airway include the use of adhesives such as mastisol and waterproof tape, as well as reinforcement with Tegaderm™ or other barriers. Given the location/field avoidance of the patient in the MRI scanner during the procedure, it is critical to note that the patient's head will be away from the anesthesiologist and will not always be accessible. A history of respiratory disorders and gastrointestinal reflux should always be evaluated since such complications could potentially affect airway securement.^21^

#### 4.1.3 Cardiac assessment

Before any anesthesia or intervention, patients with AADC deficiency should also undergo an electrocardiogram and echocardiogram, particularly since catecholamine deficiency and autonomic dysfunction can cause cardiac instability.^1^ This guidance should be applied particularly to those patients on bromocriptine who are at risk of developing cardiac fibrosis and thus at greater risk of cardiac instability under anesthesia.^1^

#### 4.1.4 Intravenous access

Peripheral intravenous (PIV) access can be extremely difficult to obtain in patients with AADC deficiency. It is unclear why PIV access is so challenging in these patients, but multiple centers have related similar experiences around this issue. Therefore, anesthesia teams should anticipate that PIV placements may need additional equipment and skilled providers. Alternatively, central access should be considered, especially if the patient is expected for a long admission or may undergo multiple interventions; either a central venous catheter or peripherally inserted central catheter (PICC) may be used. This would reduce the need for repeated PIV access for associated procedures such as follow‐up MRI and positron emission tomography scans, especially for patients with known difficult PIV placement. In most cases, PICC/central lines may be placed the day before or on the day of surgery and kept in for weeks to months.

#### 4.1.5 Glucose and medication intake

Patients should avoid prolonged fasting prior to surgery to reduce the risk and severity of hypoglycemia,^24^ and oral liquids plus glucose should be given up to 1–2 h prior to surgery, depending on the guidelines and policies of the institution. Additionally, it is recommended that patients should continue to take all prescribed medications as normal to avoid the worsening of symptoms, for example, dystonia perioperatively.

**Preoperative recommendations**

- Understand the baseline characteristics of patients to determine what interventions may be needed.
- Consider placement of a PICC/central line to circumvent potential issues with IV access for various procedures before, during, and after intracerebral gene therapy surgery.
- Encourage patients to take oral liquids plus glucose from 1 to 2 h prior to surgery to minimize the risk of hypoglycemia.

### 4.2 Intraoperative management

Patients with AADC deficiency should be cared for by a highly skilled anesthesiology team given the potential issues associated with catecholamine deficiency.^1^ There are unique challenges related to the intraoperative MRI (iMRI) setups for patients undergoing intracerebral gene therapy surgery requiring MRI guidance. These differ depending on whether an open MRI (stationary patient/stationary magnet) or movable MRI (stationary patient/mobile magnet) system is used,^25^ notably the lack of access to the patient with the open system compared with the movable system. In addition, the limited amount of MRI‐compatible equipment available may require resourceful alternatives. Regardless of these challenges, meticulous preparation is crucial to ensure patients are safe and comfortable, the endotracheal tube is secured, IV access devices are in working order, and line extensions are available. The team must be able to adapt quickly to unforeseen circumstances and with careful planning and precautionary measures, the risk of complications can be minimized. Centers taking part in intracerebral gene therapy treatment should consider conducting surgical simulations to better understand the potential challenges around process flows and issues, to help problem solve, and to devise plans ahead of time.

#### 4.2.1 Choice of anesthetic agents

Most anesthetic agents can be used in patients with AADC deficiency except for those with anti‐dopaminergic or anti‐serotonergic properties (Table 1). Neuromuscular blockers are safe to use, although non‐depolarizing neuromuscular relaxants are preferred over depolarizing agents as they are less likely to induce muscle rigidity and hyperthermia. Opioids are not usually required if the procedure is minimally invasive.

**TABLE 1:** Anesthetics and adjuvant agents for patients with AADC deficiency.

	Safe to use	To be avoided
Anesthetic agent	Propofol Ketamine Midazolam Dexmedetomidine^a^ Sevoflurane	
Neuromuscular blocker	Rocuronium Vecuronium (Sugammadex, reversal agent)	
Analgesia	Fentanyl^a^ Morphine^a^ Remifentanil^a^	Pethidine
Anti‐emetics	Dexamethasone Domperidone^b^	Ondansetron Metoclopramide Haloperidol Prochlorperazine

Abbreviation: AADC, Aromatic l‐amino acid decarboxylase.

^a^ Titrate with caution.

^b^ May be safely used in small doses as it does not cross the blood–brain barrier.

#### 4.2.2 Skin care

Patients with AADC deficiency have sensitive skin; therefore, care is needed when using adhesive tape to secure tubes/lines. The risk of skin damage should be minimized, yet taping must be sufficiently adherent to prevent tube/line displacement. This can easily happen if the tape becomes saturated with the patient's sweat or saliva and loses adhesion. While taping can be adjusted or replaced in between MRI scans during intracerebral gene therapy surgery with the movable iMRI setup, it cannot be changed if using the open iMRI setup as there is no direct access to the patient. To help protect the patient's skin from damage caused by adhesive tape, barrier cream may be applied and IV3000 dressing utilized wherever possible. Mastisol^®^ should be used with caution as it can cause skin breakdown; however, it does provide a stable adhesive to apply tape for securement of endotracheal tubes.

#### 4.2.3 Body temperature regulation

In general, anyone under general anesthesia is prone to mild hypothermia from anesthetic‐induced vasodilation and depression of hypothalamic thermoregulatory centers. Factors such as ambient temperature and IV therapy may also contribute to decreased body temperature. Patients with AADC deficiency are not only susceptible to hypothermia but also hyperthermia and diaphoresis as a result of autonomic instability due to the lack of catecholamines.^21^ Communication with the caregivers to understand how they normally manage temperature fluctuations may be helpful, but continuous monitoring of body temperature during the procedure is mandatory. Preventing hypothermia is critical, particularly for patients undergoing intracerebral gene therapy surgery with the open iMRI setup. In advance of the procedure, heating devices, infrared lamps, and blood transfusion/fluid warmers should be prepared. Patients should be prewarmed, have their extremities covered (e.g., with cotton wrap dressing) and be laid on blankets while in the MRI scanner. Conversely, prolonged exposure to the microwave effects of MRI during surgery (particularly with the open MRI setup), which may last up to 10 h, may increase the risk of hyperthermia. Where possible, ambient temperature, whether in the operating room or within the MRI scanner, should be adjusted accordingly.

#### 4.2.4 Hemodynamic stability

Hemodynamic instability is a primary concern in AADC deficiency. Therefore, anesthetic management should focus on maintaining hemodynamic stability during induction, maintenance, and emergence from anesthesia. The placement of an arterial blood pressure line can help closely monitor hemodynamic stability and guide vasopressor/inotropic therapy but is not always necessary. The authors have found that these patients vary significantly in their hemodynamic response to general anesthesia; therefore, placement of an arterial line may be determined by the clinical scenario and if the patient requires titration of inotropes for the procedure. For longer and more complicated procedures such as intracerebral gene therapy, placement of an arterial line is recommended. Any agent with anti‐dopaminergic or anti‐serotonergic properties should be avoided as they can exacerbate the symptoms of monoamine neurotransmitter deficiency. To reduce the risk of hemodynamic instability, anesthetic agents should be given at a low dose and titrated more cautiously than with the average patient as responses may vary greatly. Dexmedetomidine has been used successfully, especially for non‐invasive imaging procedures, but patients should initially be dosed with lower concentrations to assess their hemodynamic response due to the potential for an exaggerated parasympathetic response.

Preparations should be made to ensure vasopressor and inotropic support are available to manage hemodynamic instability. The first‐line treatment for hypotension in these patients is IV low‐dose dopamine (1–2 mcg/kg/min) as recommended by international consensus guidelines.^1^ However, it has been noted by the authors that, in this case series, a number of patients were unresponsive to dopamine, and our first line of treatment would be epinephrine. Norepinephrine was also used successfully. As in normal clinical practice, if a patient remains refractory to the above‐mentioned treatments, one should consider adding vasopressin. It is important to be aware that treatment of severe hypotension with pressors may increase the risk of cardiac arrhythmia and death.^26^

#### 4.2.5 Blood glucose

Glucose should be monitored while patients with AADC deficiency are under anesthesia given their increased risk of hypoglycemia. To maintain normoglycemia, dextrose (5%) may be administered where necessary.

Intraoperative recommendations

- Be mindful when securing tubes/lines with adhesive tape to reduce the risk of skin damage.
- Keep the patient as warm as possible before and during the procedure to prevent hypothermia.
- Consider using an arterial blood pressure line for patients undergoing long or complicated procedures.
- Use low doses of anesthetic agent and titrate slowly to reduce the risk of hemodynamic instability; prepare vasopressor/inotropic support in case they are needed; consider pressor availability with dopamine as the first line of inotropic support, although epinephrine or norepinephrine may also be used.
- Be aware of the limitations of the different iMRI setups for intracerebral gene therapy and be prepared to adapt quickly to unforeseen changes.

### 4.3 Postoperative disposition

#### 4.3.1 Intensive care

It is prudent to consider sending patients with AADC deficiency undergoing any lengthy procedure such as intracerebral gene therapy surgery to the intensive care unit (ICU) for postoperative care. While tracheal extubation can be done in most cases at the end of surgery, keeping the patient intubated may be considered if brain imaging is desired within the immediate postoperative period to confirm the lack of postoperative complications. Nausea and vomiting can be treated with supportive care, and corticosteroids may be used as an anti‐emetic.

#### 4.3.2 Awareness of AADC deficiency

Communication with the ICU team before and after the procedure is essential to ensure an appropriate transition of care. ICU staff should be made aware of the sympathetic impairment and the pharmacological considerations. Where appropriate, further education and information should be considered for the wider multidisciplinary team caring for patients with AADC deficiency during the postoperative period.

Postoperative recommendations

- Transfer patients to the ICU for postoperative care following intracerebral gene therapy treatment.
- Consider maintaining postoperative intubation in the ICU as needed.
- Ensure ICU staff are familiar with ways to mitigate potential post‐anesthesia complications associated with AADC deficiency.

## 5 CONCLUSION

The perioperative management of patients with AADC deficiency requires specific considerations; awareness of the lack of sympathetic autoregulation in patients is critical in providing safe anesthetic care throughout the perioperative period. A thorough preoperative assessment is necessary to identify the baseline characteristics of the patient to anticipate the need for intervention. Temperature dysregulation and hemodynamic instability are the most common challenges during anesthesia. The recent approval of gene augmentation therapy by stereotactic intracerebral delivery will increase the number of patients with AADC deficiency presenting for surgery and anesthesia. With careful planning and vigilance throughout the perioperative period, patients with AADC deficiency can safely undergo anesthesia with minimal complications.

### 5.1 Reflective questions

- What type of autonomic dysfunction can be expected in patients with AADC deficiency, and how might it impact the anesthetic management of these patients?
- What are the key components of the preoperative assessment of patients with AADC deficiency?
- What can you do perioperatively to reduce the risk of hypoglycemia in patients with AADC deficiency?

## FUNDING INFORMATION

This paper was funded by PTC Therapeutics, Inc.

## CONFLICT OF INTEREST STATEMENT

M.K.K., E.H.J., M.I., and J.N.C. have participated as advisory board members and received consulting fees from PTC Therapeutics. C.E., S.Z.F., J.L., and R.W. have nothing to disclose. The scientific team at PTC Therapeutics were given the opportunity to review the manuscript for factual accuracy but did not contribute to the content of the manuscript.

## Data Availability

Data sharing is not applicable to this article as no new data were created or analyzed in this study.

## References

[1] Wassenberg T , Molero‐Luis M , Jeltsch K , et al. Consensus guideline for the diagnosis and treatment of aromatic l‐amino acid decarboxylase (AADC) deficiency. Orphanet J Rare Dis. 2017;12(1):12. doi:10.1186/s13023-016-0522-z 28100251 PMC5241937

[2] Himmelreich N , Montioli R , Bertoldi M , et al. Aromatic amino acid decarboxylase deficiency: molecular and metabolic basis and therapeutic outlook. Mol Genet Metab. 2019;127(1):12‐22. doi:10.1016/j.ymgme.2019.03.009 30952622

[3] Civallero G , Kubaski F , Pereira D , et al. Biochemical diagnosis of aromatic‐L‐amino acid decarboxylase deficiency (AADCD) by assay of AADC activity in plasma using liquid chromatography/tandem mass spectrometry. Mol Genet Metab Rep. 2022;32:100888. doi:10.1016/j.ymgmr.2022.100888 35769135 PMC9234702

[4] Hyland K , Reott M . Prevalence of aromatic l‐amino acid decarboxylase deficiency in at‐risk populations. Pediatr Neurol. 2020;106:38‐42. doi:10.1016/j.pediatrneurol.2019.11.022 32111562

[5] Rizzi S , Spagnoli C , Frattini D , Pisani F , Fusco C . Clinical features in aromatic L‐amino acid decarboxylase (AADC) deficiency: a systematic review. Behav Neurol. 2022;2022:2210555. doi:10.1155/2022/2210555 36268467 PMC9578880

[6] Pearson TS , Gupta N , San Sebastian W , et al. Gene therapy for aromatic L‐amino acid decarboxylase deficiency by MR‐guided direct delivery of AAV2‐AADC to midbrain dopaminergic neurons. Nat Commun. 2021;12(1):4251. doi:10.1038/s41467-021-24524-8 34253733 PMC8275582

[7] Himmelreich N , Bertoldi M , Alfadhel M , et al. Prevalence of DDC genotypes in patients with aromatic L‐amino acid decarboxylase (AADC) deficiency and in silico prediction of structural protein changes. Mol Genet Metab. 2023;139(3):107624. doi:10.1016/j.ymgme.2023.107624 37348148

[8] Montioli R , Borri VC . Aromatic amino acid decarboxylase deficiency: the added value of biochemistry. Int J Mol Sci. 2021;22(6):3146. doi:10.3390/ijms22063146 33808712 PMC8003434

[9] DiBacco ML , Hinahara J , Goss TF , et al. Burden of illness in aromatic L‐amino acid decarboxylase deficiency. Ann Child Neurol Soc. 2023;1(1):75‐81. doi:10.1002/cns3.20010 PMC13359333 42563967

[10] Pearson TS , Gilbert L , Opladen T , et al. AADC deficiency from infancy to adulthood: symptoms and developmental outcome in an international cohort of 63 patients. J Inherit Metab Dis. 2020;43(5):1121‐1130. doi:10.1002/jimd.12247 32369189 PMC7540529

[11] Lee LK , Cheung KM , Cheng WW , et al. A rare cause of severe diarrhoea diagnosed by urine metabolic screening: aromatic L‐amino acid decarboxylase deficiency. Hong Kong Med J. 2014;20(2):161‐164. doi:10.12809/hkmj133922 24714172

[12] Chen PW , Lee NC , Chien YH , Wu JY , Wang PC , Hwu WL . Diagnosis of aromatic L‐amino acid decarboxylase deficiency by measuring 3‐O‐methyldopa concentrations in dried blood spots. Clin Chim Acta. 2014;431:19‐22. doi:10.1016/j.cca.2014.01.034 24513538

[13] Chien YH , Lee NC , Tseng SH , et al. Efficacy and safety of AAV2 gene therapy in children with aromatic L‐amino acid decarboxylase deficiency: an open‐label, phase 1/2 trial. Lancet Child Adolesc Health. 2017;1(4):265‐273. doi:10.1016/S2352-4642(17)30125-6 30169182

[14] Berqkvist M , Stephens C , Schilling T , et al. Aromatic L‐amino acid decarboxylase deficiency: a systematic review. Future Neurol. 2022;17(4):FNL63. doi:10.2217/fnl-2022-0012

[15] Brun L , Ngu LH , Keng WT , et al. Clinical and biochemical features of aromatic L‐amino acid decarboxylase deficiency. Neurology. 2010;75(1):64‐71. doi:10.1212/WNL.0b013e3181e620ae 20505134

[16] Anguela XM , High KA . Entering the modern era of gene therapy. Annu Rev Med. 2019;70:273‐288. doi:10.1146/annurev-med-012017-043332 30477394

[17] Simons CL , Hwu WL , Zhang R , Simons MJHG , Bergkvist M , Bennison C . Long‐term outcomes of eladocagene exuparvovec compared with standard of care in aromatic L‐amino acid decarboxylase (AADC) deficiency: a modelling study. Adv Ther. 2023;40(12):5399‐5414. doi:10.1007/s12325-023-02689-6 37803205 PMC10611606

[18] Tai CH , Lee NC , Chien YH , et al. Long‐term efficacy and safety of eladocagene exuparvovec in patients with AADC deficiency. Mol Ther. 2022;30(2):509‐518. doi:10.1016/j.ymthe.2021.11.005 34763085 PMC8822132

[19] Roubertie A , Opladen T , Brennenstuhl H , et al. Gene therapy for aromatic L‐amino acid decarboxylase deficiency: requirements for safe application and knowledge‐generating follow‐up. J Inherit Metab Dis. 2023;47:463‐475. doi:10.1002/jimd.12649 37402126

[20] Kojima K , Nakajima T , Taga N , et al. Gene therapy improves motor and mental function of aromatic l‐amino acid decarboxylase deficiency. Brain. 2019;142(2):322‐333. doi:10.1093/brain/awy331 30689738 PMC6377184

[21] McCarthy A , Black C . Anaesthesia management of a child with aromatic L‐amino acid decarboxylase deficiency. Anaesth Rep. 2022;10(1):e12152. doi:10.1002/anr3.12152 35233534 PMC8861587

[22] Vutskits L , Menache C , Manzano S , et al. Anesthesia management in a young child with aromatic l‐amino acid decarboxylase deficiency. Paediatr Anaesth. 2006;16(1):82‐84. doi:10.1111/j.1460-9592.2005.01605.x 16409536

[23] Wu JJ , Chuang YH , Wu CY . Feasibility and safety of using supraglottic airway devices for pediatric patients undergoing magnetic resonance imaging: a case series of high‐risk patients. Asian J Anesthesiol. 2021;59(3):96‐101. doi:10.6859/aja.202109_59(3).0003 34266229

[24] Arnoux JB , Damaj L , Napuri S , et al. Aromatic L‐amino acid decarboxylase deficiency is a cause of long‐fasting hypoglycemia. J Clin Endocrinol Metab. 2013;98(11):4279‐4284. doi:10.1210/jc.2013-2740 24037885

[25] McClain CD , Rockoff MA , Soriano SG . Anesthetic concerns for pediatric patients in an intraoperative MRI suite. Curr Opin Anaesthesiol. 2011;24(5):480‐486. doi:10.1097/ACO.0b013e32834ab4e3 21841476

[26] Anselm IA , Darras BT . Catecholamine toxicity in aromatic L‐amino acid decarboxylase deficiency. Pediatr Neurol. 2006;35(2):142‐144. doi:10.1016/j.pediatrneurol.2006.01.008 16876014

